# Supplementary material for: Global hypo-methylation in a proportion of glioblastoma enriched for an astrocytic signature is associated with increased invasion and altered immune landscape
Source: eLife. 2022 Nov 22;11:e77335. doi: 10.7554/eLife.77335 (PMC9681209; doi:10.7554/eLife.77335)
Supplement: Figure 2—source data 1. [file elife-77335-fig2-data1.zip › Figure_2_source_data_1/Figure_2I_J/homerResults/motif20.similar.html]

motif20

## Information for motif20

C
G
T
A
G
T
C
A
T
G
C
A
C
T
G
A
A
C
G
T
A
C
G
T
C
G
T
A
G
T
A
C
C
G
A
T
C
G
A
T
  
Reverse Opposite:  

G
C
T
A
G
C
T
A
C
A
T
G
C
G
A
T
G
T
C
A
C
G
T
A
A
G
C
T
A
C
G
T
C
A
G
T
G
C
A
T
  

|  |  |
| --- | --- |
| p-value: | 1e-34 |
| log p-value: | -7.879e+01 |
| Information Content per bp: | 1.705 |
| Number of Target Sequences with motif | 41.0 |
| Percentage of Target Sequences with motif | 5.65% |
| Number of Background Sequences with motif | 1.3 |
| Percentage of Background Sequences with motif | 0.47% |
| Average Position of motif in Targets | 114.7 +/- 50.8bp |
| Average Position of motif in Background | 144.0 +/- 6.7bp |
| Strand Bias (log2 ratio + to - strand density) | 0.3 |
| Multiplicity (# of sites on avg that occur together) | 1.17 |
| Motif File: | file (matrix) reverse opposite |

### Similar de novo motifs found

|  |  |  |  |  |  |  |  |
| --- | --- | --- | --- | --- | --- | --- | --- |
| Rank | Match Score | Redundant Motif | P-value | log P-value | % of Targets | % of Background | Motif file |
| 1 | 0.866 | T G C A G T A C C A T G C A G T G C T A T C G A G A C T C A G T | 1e-29 | -68.322589 | 6.61% | 1.01% | motif file (matrix) |
| 2 | 0.641 | T C A G C T G A T C G A G T C A C G A T C A G T G C A T G A T C G C T A A C G T A C G T T G C A C G T A T C G A C G T A | 1e-17 | -40.382795 | 4.82% | 0.87% | motif file (matrix) |
| 3 | 0.882 | C G T A C G T A A C G T A C G T C G T A A G T C | 1e-15 | -36.651565 | 8.54% | 2.74% | motif file (matrix) |
| 4 | 0.620 | C A G T C G A T C T A G C G T A G T C A G T C A A G C T C A G T C G T A G A C T | 1e-14 | -32.596834 | 3.17% | 0.41% | motif file (matrix) |
